# Association of MMP-9 and TIMP-1 concentration with neurological outcome after cardiac arrest and resuscitation – a substudy of the COMACARE trial

**DOI:** 10.1016/j.resplu.2025.101187

**Published:** 2025-12-05

**Authors:** Emilia Kortesuo, Joonas Tirkkonen, Matti Reinikainen, Pirkka T. Pekkarinen, Eeva Moilanen, Liisa Petäjä, Markus B Skrifvars, Johanna Hästbacka

**Affiliations:** a Tampere University, Faculty of Medicine and Health Technology, Tampere, Finland; b Department of Anaesthesia and Intensive Care, Tampere University Hospital, Wellbeing Services County of Pirkanmaa, Tampere, Finland; c Department of Anaesthesiology and Intensive Care, Kuopio University Hospital and University of Eastern Finland, Kuopio, Finland; d Department of Anaesthesia and Intensive Care Medicine, Helsinki University Hospital and University of Helsinki, Finland; e The Immunopharmacology Research Group, Faculty of Medicine and Health Technology, Tampere University and Tampere University Hospital, Tampere, Finland

**Keywords:** Out-of-hospital cardiac arrest, OHCA, MMP-9, TIMP-1, Inflammation

## Abstract

**Background and aim:**

Out-of-hospital cardiac arrest (OHCA) leads to an inflammatory response, including alterations in matrix metalloproteinase (MMP)-9 and tissue inhibitor of matrix metalloproteinase (TIMP)-1 concentrations. We investigated whether the MMP-9 and TIMP-1 plasma concentrations of OHCA patients are elevated and associated with outcome among OHCA patients.

**Methods:**

This was a substudy of the prospective COMACARE trial (NCT02698917). We included 112 OHCA patients and measured MMP-9 and TIMP-1 concentrations at ICU admission, 24, 48 and at 72 h. Preoperative blood samples from 40 age- and sex-matched elective cardiac surgery patients were used as controls. We defined favourable outcome as a Cerebral Performance Category (CPC) 1–2 at six months.

**Results:**

The median (interquartile range) MMP-9 concentrations at admission for OHCA patients and for controls were 369 (228–619) ng/mL and 66 (41–114) ng/mL, respectively, *p* < 0.001. The TIMP-1 concentrations for OHCA patients at admission and for controls were 137 (104–163) ng/mL and 79 (71–96) ng/mL, respectively, *p* < 0.001. The MMP-9 levels peaked at admission; 448 (241–700) ng/mL in patients with CPC ≥ 3 as compared with 340 (224–563) ng/mL in patients with CPC 1–2 (*p* = 0.103). TIMP-1 concentrations peaked at 48 h; 223 (174–323) ng/mL in patients with CPC ≥ 3 as compared with 201 (148–273) ng/mL in patients with CPC 1–2 (*p* = 0.104). In a logistic regression model, neither biomarker demonstrated association with outcome.

**Conclusion:**

OHCA patients had higher plasma concentrations of MMP-9 and TIMP-1 than elective surgery patients. However, the concentrations showed no association with outcome.

## Introduction

Cardiac arrest leads to sudden loss of circulation and causes ischemia to which especially the central nervous system is sensitive.^1^ After successful resuscitation from cardiac arrest, a multi-organ dysfunction called post-cardiac arrest syndrome (PCAS) occurs in some patients.^2^ It is likely caused by multiple factors, but inflammatory response resembling septic shock, following whole-body ischemia and reperfusion at return of spontaneous circulation (ROSC), plays a substantial part.^3^ Therefore, inflammatory biomarkers are interesting regarding the development of organ dysfunction and brain injury.^4^, ^5^

After out-of-hospital cardiac arrest (OHCA), blood concentrations of matrix metalloproteinase (MMP)-9 are increased,^6^ but their role has not been properly investigated. In a previous study, MMP-9 and TIMP-1 concentrations were compared between patients treated with therapeutic hypothermia and a control group without targeted temperature management.^6^ The main finding was that MMP-9 levels decreased during hypothermia but increased again after rewarming. However, only two sampling time points were available, and no admission samples were collected. In addition, the study lacked a control group with underlying cardiovascular pathology, which would have been important for assessing the magnitude of MMP-9 and TIMP-1 changes during acute global ischaemia.^6^ MMPs are a group of zinc-dependent endopeptidases with collagen cleaving capability involved in the breakdown and remodelling of tissue in homeostatic as well as pathologic processes.^7^ MMP-9 is a gelatinase belonging to the metalloproteinase family and it is found in low levels in normal brain tissue, but released into circulation in a proenzyme form from leukocytes, neurons and glial cells upon stimuli such as ischemia.^8^ The expression and activity of MMP-9 is regulated by tissue inhibitor of matrix metalloproteinase (TIMP)-1.^9^ In an experimental model of stroke and traumatic brain injury in mice, overexpression of TIMP-1 has been shown to limit the extent of ischemic injury, suggesting inhibition of MMP-9 to be beneficial,^10^ which is pharmacologically possible.^11^, ^12^

Elevated MMP-9 concentrations have been associated with the development of atherosclerosis.^13^ To extend the knowledge on the temporal evolution of MMP-9 and TIMP-1 concentrations after cardiac arrest, we analysed MMP-9 and TIMP-1 concentrations from admission and compared the levels between OHCA patients and patients with cardiovascular disease burden without cardiac arrest. For comparison, we used preoperative plasma samples from patients scheduled for elective cardiac surgery. The aim of this study was to investigate whether plasma MMP-9 and TIMP-1 concentrations are elevated in OHCA patient compared to controls, and how MMP-9 and TIMP-1 concentrations are associated with neurological outcome in patients resuscitated from OHCA. Additionally, we wanted to observe the progression of MMP-9 and TIMP-1 over time to address the existing knowledge gap regarding their temporal dynamics and the magnitude of the change in the acute critical illness after global ischaemia due to OHCA.

## Materials and methods

We used the Strengthening the reporting of observational studies in epidemiology (STROBE) guideline in reporting the results of the study.^14^

### Study setting

Our study is a substudy of the COMACARE trial (NCT02698917).^15^, ^16^, ^17^ In brief, the study included 18–80-year-old OHCA patients admitted to the participating ICUs with a time from collapse to return of spontaneous circulation (ROSC) of 10–45 min and ventricular fibrillation/tachycardia as an initial rhythm. Patients were unconscious (Glasgow Coma Scale (GCS) < 5) and mechanically ventilated. The patients were treated with a targeted temperature of 33 or 36 °C for 24 h. Patients were randomised into eight treatment groups with different treatment combinations of low normal or high normal PaCO_2_ (4.5–4.7 kPa and 5.8–6 kPa, respectively), mean arterial pressure (MAP 65–75 mmHg and 80–100 mmHg, respectively), and normoxia (PaO_2_ 10–15 kPa) or moderate hyperoxia (PaO_2_ 20–25 kPa). Patients were recruited between March 2016 and November 2017. The elective surgery control patients were matched regarding age and sex to the OHCA patients.

### Ethics

This study was conducted according to the principles of the Declaration of Helsinki. After eligibility screening, written informed consent was obtained from the next-of-kin with a deferred consent when necessary. The COMACARE study protocol and the amendment approving the current analysis were approved by the Research Ethics Committee of the Northern Savo Hospital District (295/13.02.00/2015 §53). The 40 comparator samples were obtained from patients participating in the single-centre prospective observational FINNAKI-HEART study,^18^ approved in The Ethics Committee of Helsinki University Hospital (§124 31.8.2011 and § 24 Dnro 18/13/03/02/2010). The control patients were recruited between September 2011 and June 2012.

### Laboratory analyses

We collected MMP-9 and TIMP-1 blood samples at admission and at 24, 48 and 72 h after OHCA. After obtaining the blood samples from the arterial line, they were centrifuged (2000 G, 10 min) and stored at −70 °C until analysis. Blood samples from the controls were collected from arterial cannulas before anaesthesia induction, plasma was separated, and the samples were stored at −80 °C until analysis.^18^ Plasma concentrations of MMP-9 and TIMP-1 (detection limit 7.8 pg/ml for both) were measured by Enzyme-Linked Immunosorbent Assay (ELISA) using reagents from R&D Systems Europe Ltd, Abingdon, UK. The inter-assay coefficient of variation was 3.4 % for MMP-9 and 3.3 % for TIMP-1.

### Outcomes

The neurological outcome was assessed using the Cerebral Performance Category (CPC) at six months after cardiac arrest by an experienced neurologist blinded to the intervention groups. A CPC score of 1–2 at six months was categorised as a favourable neurological outcome, while a score of 3–5 was considered an unfavourable neurological outcome.^19^

### Statistical methods

We used SPSS statistics version 29.0 (IB, Armonk, NY, USA) to perform the analyses. The data are presented as medians with interquartile ranges (IQR). We assessed visually the Gaussian distribution profile. To evaluate between-group differences, we used the Mann-Whitney *U* test for continuous variables and Chi-Square test or Fisheŕs exact test for categorical variables. We considered a *p*-value of <0.05 as statistically significant. We also calculated the molar ratio of MMP-9 and TIMP-1. Boxplot diagrams were transformed to a logarithmic scale. We tested the independent association of MMP-9 and TIMP-1 for neurological outcome using logistic regression models adjusted for confounding variables. For MMP-9 and TIMP-1, we selected timepoints that corresponded to the highest median plasma concentrations (admission for MMP-9 and the 48-h timepoint for TIMP-1). We assessed age, bystander resuscitation (resuscitation vs. no resuscitation), Acute Physiology And Chronic Health Evaluation (APACHE) II score^20^ without age points and time to ROSC in univariable logistic regression as potential confounders. We included variables that were associated with the outcome with a *p*-value less than 0.1 in the final models.

## Results

**Fig. 1:**
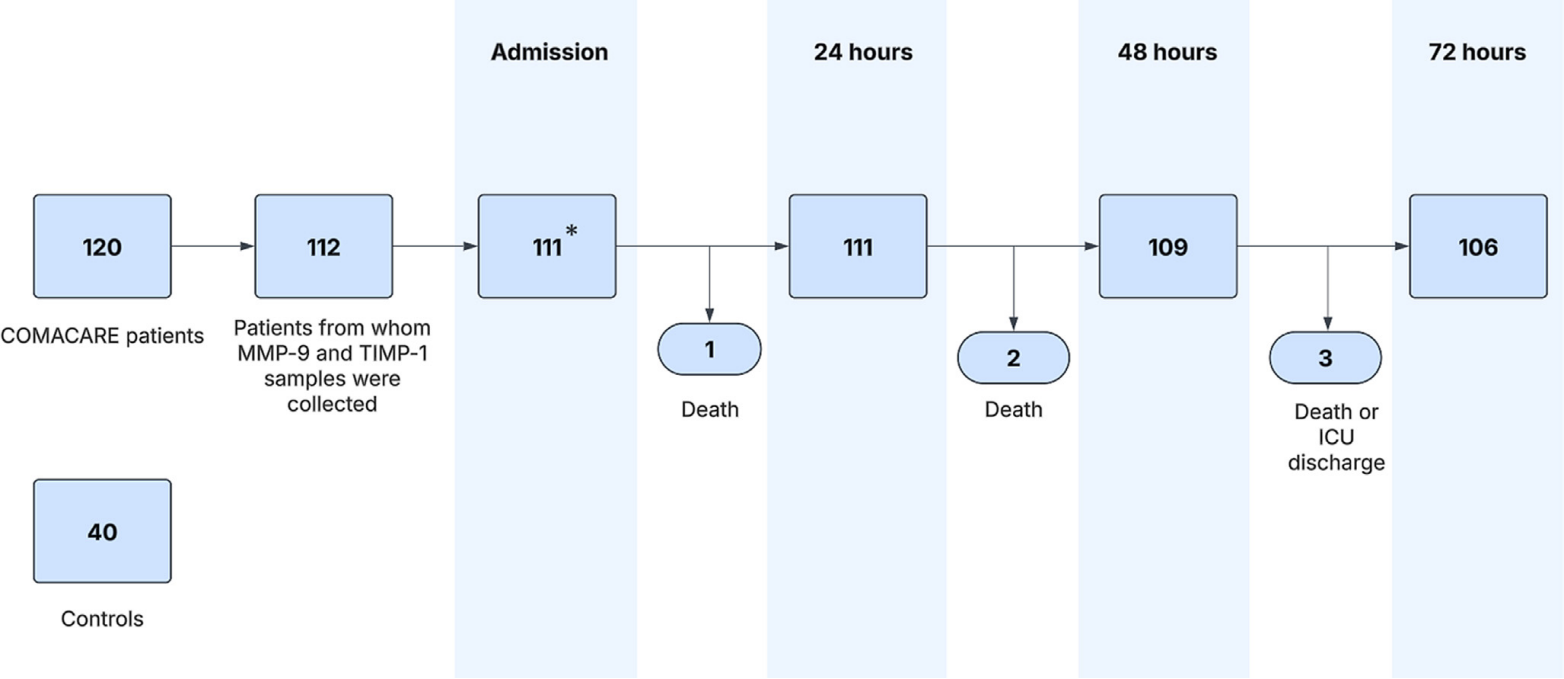
**Description of patient flow**. *Admission sample was missing for one patient. Matrix metalloproteinase (MMP), tissue inhibitor of matrix metalloproteinase (TIMP), intensive care unit (ICU).

Fig. 1 presents the patient flow. Baseline characteristics of patients are shown in Table 1. The OHCA patients had significantly higher MMP-9, and TIMP-1 concentrations compared to controls. Of the control patients, 75 % (*n* = 30) had coronary artery disease.

**Table 1:** Characteristics of the cardiac arrest patients and control patients.

	**Cardiac arrest, *n* = 112**	**Controls, *n* = 40**	***p* value**
Age	62 (53–68)	62 (52–69)	0.922
Sex male, *n* (%)	92 (82)	34 (85)	0.680
APACHE II	28 (24–31)	−	
Bystander resuscitation, *n* (%)	93 (83)	−	
Time to ROSC (min)	21 (16–26)	−	
Target temperature 33 °C *n* (%)	75 (67)	−	
Admission MMP-9 ng/mL	369 (228–619)	66 (41–114)	<0.001
Admission TIMP-1 ng/mL	137 (104–163)	79 (71–96)	<0.001

Continuous data are presented as medians (interquartile range). Acute Physiology And Chronic Health Evaluation (APACHE), return of spontaneous circulation (ROSC), matrix metalloproteinase (MMP), tissue inhibitor of matrix metalloproteinase (TIMP), interquartile range (IQR).

**Fig. 2:**
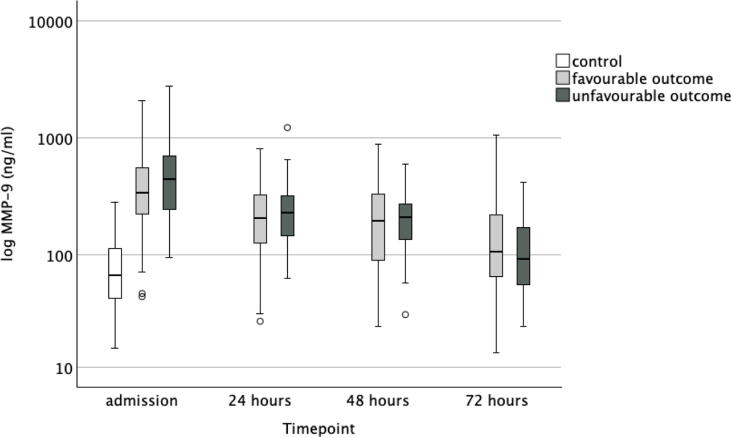
**Concentrations of matrix metalloproteinase (MMP)-9 in control patients and in cardiac arrest patients with favourable outcome and in those with unfavourable outcome**.

**Fig. 3:**
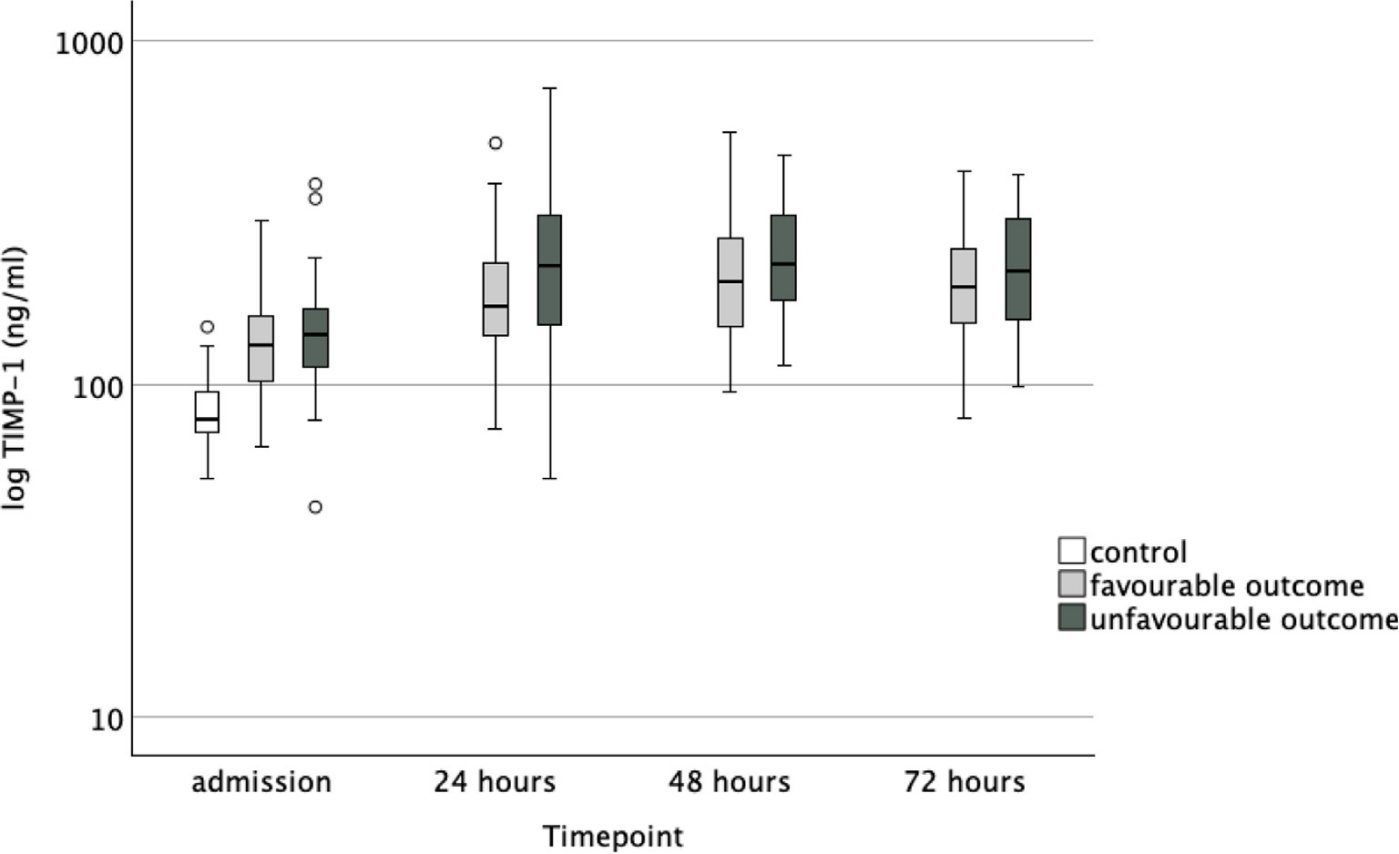
**Concentrations of tissue inhibitor of matrix metalloproteinase (TIMP)-1 in control patients and in cardiac arrest patients with favourable outcome and in those with unfavourable outcome**.

**Fig. 4:**
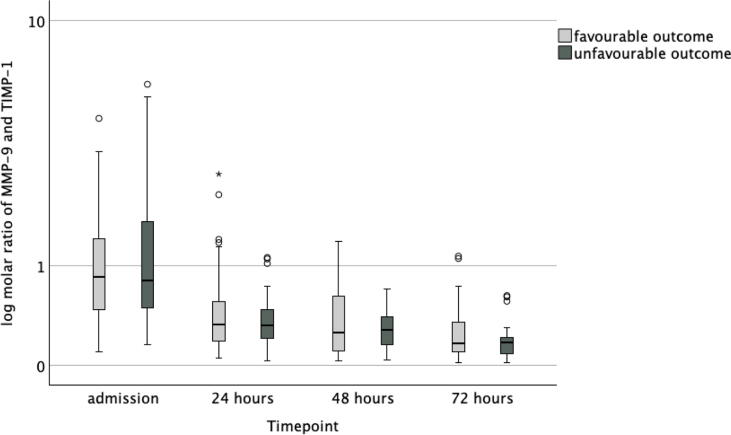
**Molar ratio of matrix metalloproteinase (MMP)-9 and tissue inhibitor of matrix metalloproteinase (TIMP)-1 in cardiac arrest patients with favourable outcome and in those with unfavourable outcome**.

Six months after cardiac arrest, 53 (48 %) patients had a CPC 1 (good cerebral performance), 20 (18 %) patients had a CPC 2 (moderate cerebral performance), 2 (2 %) patients had a CPC 3 (severe cerebral disability), none had a CPC 4 (vegetative state) and 36 (32 %) had a CPC 5 (death). Data on neurological outcome were missing for one patient. When comparing neurological outcome groups as dichotomised to favourable (CPC 1–2) and unfavourable (3–5), there was a significant difference in age, time to ROSC, bystander resuscitation, proportion of patients treated in the target temperature of 33 °C, and APACHE II score. In Table 2, we present the data according to groups indexed by outcome. There was no significant difference in MMP-9 concentrations between patients with favourable and those with unfavourable neurological outcomes at any timepoint. Neither were there any significant difference in TIMP-1 concentrations between outcome groups at admission, at 48 h or at 72 h. At 24-h timepoint, the median (IQR) TIMP-1 concentrations in the favourable and unfavourable neurological outcome groups were 169 (139–229) ng/mL and 222 (149–314) ng/mL, respectively (*p* = 0.024). There was no significant difference in the molar ratio between outcome groups. The temporal progression of MMP-9 and TIMP-1 levels and the molar ratio of MMP-9 and TIMP-1 are presented in Fig. 2, Fig. 3, Fig. 4.

**Table 2:** Characteristics of the neurological outcome groups.

	**Favourable outcome**	**Unfavourable outcome**	***p* value**
*n* (%)	73 (66)	38 (34)	
Age	58 (51–66)	66 (58–74)	0.006
Sex male *n* (%)	61 (84)	30 (79)	0.548
BMI	26 (24–29)	26 (23–29)	0.738
APACHE II	27 (24–29)	31 (26–35)	<0.001
PCI *n* (%)	36 (49)	15 (40)	0.263
NYHA class *n* (%)			0.368
1	59 (82)	29 (78)	
2	9 (13)	4 (11)	
3	4 (6)	2 (5)	
4	0 (0)	2 (5)	
Bystander resuscitation *n* (%)	66 (90)	27 (71)	0.026
Time to ROSC (min)	17 (15–22)	26 (22–32)	<0.001
Target temperature 33 °C *n* (%)	56 (77)	19 (50)	0.004
**MMP-9, ng/mL**			
Admission	340 (224–563)	448 (241–700)	0.103
24 h	206 (125–329)	231 (139–321)	0.442
48 h	196 (88–333)	210 (131–272)	0.644
72 h	107 (62–222)	93 (54–177)	0.421
**TIMP-1, ng/mL**			
Admission	130 (102–160)	140 (111–170)	0.113
24 h	169 (139–229)	222 (149–314)	0.024
48 h	201 (148–273)	223 (174–323)	0.104
72 h	193 (150–249)	214 (153–308)	0.234
**Molar ratio of MMP-9 and TIMP-1**			
Admission	0.86 (0.47–1.42)	0.80 (0.48–1.74)	0.439
24 h	0.33 (0.19–0.56)	0.32 (0.20–0.47)	0.563
48 h	0.26 (0.11–0.64)	0.28 (0.16–0.41)	0.966
72 h	0.17 (0.10–0.36)	0.17 (0.08–0.22)	0.275

Continuous data are presented as medians (interquartile range). Acute Physiology And Chronic Health Evaluation (APACHE) Return of spontaneous circulation (ROSC), percutaneous coronary intervention (PCI) matrix metalloproteinase (MMP), tissue inhibitor of matrix metalloproteinase (TIMP), interquartile range (IQR).

In a logistic regression model, neither MMP-9 nor TIMP-1 were independently associated with unfavourable neurological outcome at 6 months when adjusted for relevant confounders. In these analyses, we used the biomarker concentrations from those timepoints when the median concentration was at its highest. The results are presented in Table 3, Table 4.

**Table 3:** Logistic regression model of matrix metalloproteinase (MMP)-9 for association with a 6-month unfavourable neurological outcome.

	**OR**	**95 % CI**	***p*-value**
Age (year)	1.08	1.02–1.13	0.007
APACHE II score (without age points)	1.03	0.90–1.18	0.677
Bystander resuscitation (No)	5.91	1.31–26.60	0.021
Delay to ROSC (min)	1.25	1.13–1.37	<0.001
MMP-9 concentration at admission (ng/mL)	1.00	1.00–1.00	0.076

Acute Physiology And Chronic Health Evaluation (APACHE), return of spontaneous circulation (ROSC), odds ratio (OR), confidence interval (CI).

**Table 4:** Logistic regression model for tissue inhibitor of matrix metalloproteinase (TIMP)-1 for association with a 6-month unfavourable neurological outcome.

	**OR**	**95 % CI**	***p*-value**
Age (year)	1.05	1.01–1.10	0.031
APACHE II score (without age points)	1.08	0.96–1.22	0.205
Bystander resuscitation (No)	3.94	0.97–15.93	0.055
Delay to ROSC (min)	1.19	1.09–1.29	<0.001
TIMP-1 concentration at 48-h timepoint (ng/mL)	1.00	1.00–1.01	0.669

Acute Physiology And Chronic Health Evaluation (APACHE), return of spontaneous circulation (ROSC), odds ratio (OR), confidence interval (CI).

## Discussion

In this post-hoc laboratory substudy of the COMACARE trial, we found that MMP-9 and TIMP-1 concentrations were significantly higher in OHCA patients compared to elective cardiac surgery patients. MMP-9 peaked at ICU admission and TIMP-1 peaked at 48 h after OHCA. However, there was no significant difference in MMP-9 concentrations or TIMP-1 concentrations, except at one time point, between OHCA patients with favourable outcome and those with unfavourable outcome. Neither was there any significant difference in the molar ratio of MMP-9 and TIMP-1 at any timepoint between the outcome groups. In a logistic regression analysis, neither MMP-9 nor TIMP-1 was independently associated with unfavourable neurological outcome when adjusted for relevant confounders.

We chose elective cardiac surgery patients as control patients to gain understanding of the assumed baseline blood concentrations of MMP-9 and TIMP-1, since alterations in their concentrations have been associated with atherosclerosis and coronary artery disease (CAD), diagnoses common among OHCA patients.^21^ However, the levels of MMP-9 and TIMP-1 appear to be significantly higher in OHCA patients. MMP-9 plasma concentration has been recognised as an indicator of cardiovascular mortality in patients with CAD,^22^ and TIMP-1 has also been identified as an independent predictor of cardiovascular events and cardiac mortality.^23^

Disruption of the blood–brain barrier (BBB) is a key event leading to secondary brain damage, and elevated MMP-9 concentrations have been found to be associated with this process after ischemic stroke.^24^ In the breakdown of the BBB, MMP-9 degrades tight junctions of the endothelial basement membrane, thereby allowing inflammatory cells to migrate through the BBB. This is highlighted by a study conducted on MMP-9 depleted mice, which showed less injury to the BBB and a reduced degree of brain injury after an ischemic insult^25^ but in our study, we failed to demonstrate any association between MMP-9 and long-term functional outcome in OHCA patients. The significant finding in TIMP-1 24-h concentrations might be due to chance, or it may indicate a transient but biologically relevant increase of TIMP-1. As functional outcome is mainly understood as an endpoint reflecting neurological recovery after OHCA, other organ systems may also be involved more or less directly in these patients. Interestingly, it has previously been shown that MMP-9 concentrations are elevated during heart surgery.^26^ In addition, in patients with myocardial infarction, MMP-9 has been shown to be associated with cardiac remodelling and outcome.^27^ This should be considered when interpreting our results, since a considerable proportion of OHCA are caused by acute myocardial ischaemia or infarction, and patients may also have undergone emergency heart surgery. In our study, MMP-9 and TIMP-1 showed different kinetics, with earlier upregulation of MMP-9 compared to TIMP-1, which peaked at 48 h after admission. MMP-9 is rapidly released from neutrophil granules upon inflammatory stimuli^28^ but it also has de novo synthesis that affects its concentrations later.^29^ The different kinetics of MMP-9 and TIMP-1 in our study may also be explained by a previous observation that hypothermia transiently decreases MMP-9 concentrations,^6^ while the concentration of TIMP-1 seemed less affected by hypothermia. The patients in our study were mainly treated with therapeutic hypothermia.

In patients resuscitated from OHCA, the inflammatory response is not limited to the brain, but a generalised inflammatory process occurs.^3^ Alterations in MMP-9 and TIMP-1 concentrations are also observed in other types of inflammatory states. A substantial number of patients develop infections after OHCA.^30^, ^31^ The most common infection after OHCA is pneumonia^32^ which in itself raises MMP-9 levels.^33^ Additionally, acute coronary syndrome, which is often a cause of OHCA,^34^ also elevates MMP-9 and TIMP-1 concentrations,^35^ Considering the aforementioned factors, OHCA alone might not explain all the alterations in MMP-9 and TIMP-1 concentrations that we observed.

Although we found no association between MMP-9 or TIMP-1 concentrations and outcome, the topic remains of interest for future studies. We observed a trend toward higher concentrations of both biomarkers in patients with unfavourable outcomes. However, the dichotomous classification into favourable and unfavourable outcomes is a crude measure, and the number of patients with an unfavourable outcome was small. Furthermore, the data in this study are from patients treated with therapeutic hypothermia. A future study in the current targeted temperature management era, with a larger, focused patient population is needed to confirm or refute our findings. If a significant association of MMP-9 with a patient-important outcome were confirmed, studies on pharmacological inhibition of MMP-9 would be an interesting next step.

### Strengths and limitations

This was a substudy of a randomised controlled trial conducted in a government-funded healthcare system, and the patient outcome assessor was blinded. Blood samples were collected consistently, and MMP-9 and TIMP-1 values were analysed after the whole patient population was recruited, and therefore did not affect the outcome assessment. There are also limitations. We only included patients with a shockable initial rhythm (VF/VT), so the findings cannot be generalised to all cardiac arrest patients. The statistical power of the study is limited since the number of patients is small and there were only 38 patients with an unfavourable outcome. We acknowledge that the small sample size involves a risk of type II error (false negative). This was an exploratory substudy, and we did not perform a priori power calculation. Since we had five variables entered per 38 outcomes in the logistic regression models, the possibility of overfitting should be considered.^36^ Additionally, we only had limited data on the control patients, so there is a possibility that the groups are not completely comparable. The COMACARE study was conducted between 2016 and 2017, and the results are not fully generalisable to the present post-OHCA management in the ICU^37^ as some of the samples were collected at a centre where targeted temperature management at 33 °C was common, which may have had a lowering effect on the MMP-9 levels.^6^ The cryopreservation time between patients and controls differ, but MMP-9 has been shown to remain stable over an extended period when stored at appropriate temperatures.^38^

## Conclusion

We found that plasma concentrations of MMP-9 and TIMP-1 were elevated in OHCA patients compared to elective cardiac surgery patients. However, there was no significant difference in MMP-9 or TIMP-1 levels between the patients with favourable outcome and those with unfavourable outcome. Neither MMP-9 nor TIMP-1 was independently associated with an unfavourable outcome.

## CRediT authorship contribution statement

**Emilia Kortesuo:** Writing – review & editing, Writing – original draft, Visualization, Investigation, Formal analysis. **Joonas Tirkkonen:** Writing – review & editing, Supervision. **Matti Reinikainen:** Writing – review & editing, Resources. **Pirkka T. Pekkarinen:** Writing – review & editing. **Eeva Moilanen:** Writing – review & editing, Resources, Methodology. **Liisa Petäjä:** Writing – review & editing, Resources. **Markus B Skrifvars:** Writing – review & editing, Resources, Funding acquisition. **Johanna Hästbacka:** Writing – review & editing, Writing – original draft, Supervision, Resources, Project administration, Methodology, Investigation, Data curation, Conceptualization.

## Funding

This work was supported by Finska Läkaresällskapet Sigrid Juselius Stiftelse and Competitive Research Funding (VTR Funding) of Tampere University Hospital, Tampere, Finland.

## Declaration of competing interest

The authors declare the following financial interests/personal relationships which may be considered as potential competing interests: Markus Skrifvars reports financial support was provided by Finska Läkaresällskapet, Sigrid Juselius Stiftelse. Eeva Moilanen reports financial support was provided by Competitive Reseach Funding (VTR Funding) of Tampere University Hospital, Tampere, Finland. Markus Skrifvars reports a relationship with International Liaison Committee on Resuscitation, ALS Task Force member that includes:. Markus Skrifvars reports a relationship with Resuscitation journal, Editorial Board that includes: board membership. If there are other authors, they declare that they have no known competing financial interests or personal relationships that could have appeared to influence the work reported in this paper.
